# Evaluation of differential renal function in children – a comparative study between magnetic resonance urography and dynamic renal scintigraphy

**DOI:** 10.1186/s12887-024-04694-2

**Published:** 2024-03-25

**Authors:** Małgorzata Gołuch, Agnieszka Pytlewska, Jędrzej Sarnecki, Paulina Chodnicka, Anna Śliwińska, Łukasz Obrycki, Elżbieta Jurkiewicz

**Affiliations:** 1 Department of Diagnostic Imaging, The Children’s Memorial Health Institute, Warsaw, Poland; 2 Department of Radiology, University Hospital Basel, Basel, Switzerland; 3 Department of Nuclear Medicine, The Children’s Memorial Health Institute, Warsaw, Poland; 4 Department of Nephrology, Kidney Transplantation and Hypertension, The Children’s Memorial Health Institute, Warsaw, Poland

**Keywords:** Magnetic resonance urography, Differential renal function, Dynamic renal scintigraphy, Pelvicalyceal dilatation, CAKUT, Pediatric urology, Urinary system, Functional imaging

## Abstract

**Background:**

Urinary system anomalies, both congenital and acquired, constitute a relatively common clinical problem in children. The main role of diagnostic imaging is to determine early diagnosis and support therapeutic decisions to prevent the development of chronic renal disease. The aim of this study was to evaluate the utility of magnetic resonance urography (MRU) in assessment of urinary system in children, by comparing differential renal function calculated using MRU with dynamic renal scintigraphy (DRS).

**Materials and methods:**

The study group consisted of 46 patients aged 1 week to 17 years (median 7 (0.5; 13) years, 17 (37%) girls, 29 (63%) boys), who underwent dynamic renal scintigraphy due to various clinical reasons. All participants underwent MRU, which was used to measure differential renal function. Functional analysis was performed using dedicated external software (CHOP-fMRU and pMRI without prior knowledge of DRS results. MRU results acquired using pMRI were assessed for inter and intraobserver agreement.

**Results:**

Statistical analysis of the results showed excellent agreement between MRU and DRS in measuring differential renal function with Pearson correlation coefficient 0.987 for CHOP-fMRU and 0.971 for pMRI, *p* < 0.001. Interclass correlation coefficient (ICC) for these programs was 0.987 (95% CI 0.976–0.993) and 0.969 (95% CI 0.945–0.983) respectively, *p* < 0.001. The Bland-Altman 95% limits of agreement for CHOP-fMRU results vs. DRS was − 6.29–5.50 p.p. and for pMRI results vs. DRS − 9.15–9.63 p.p. The differential renal function measurements calculated in pMRI showed excellent intraobserver and interobserver agreement with ICC 0.996 (95% CI 0.994–0.998) and 0.992 (95% CI 0.986–0.996) respectively, *p* < 0.001.

**Conclusions:**

The study showed no significant differences between magnetic resonance urography and dynamic renal scintigraphy in calculating differential renal function. It indicates high utility of MRU in the evaluation of urinary system in children.

## Background

Urinary system anomalies are relatively common among children and constitute an important clinical problem due to the risk of chronic kidney disease development. Congenital anomalies of kidney and urinary tract (CAKUT) are the main cause of chronic kidney disease in children [1] being responsible for 48% of cases in developed countries [2]. Moreover a history of clinically evident kidney disease in childhood (including CAKUT, pyelonephritis and glomerular disease), even if renal function was normal in adolescence, is proved to be associated with increased risk of chronic kidney disease and end-stage renal disease in adults, suggesting that any kidney injury may have long-term consequences [3]. 

Therefore accurate imaging of urinary system is crucial as it facilitates an early diagnosis and the choice of optimal treatment [4]. Magnetic resonance urography (MRU) is a unique method that allows a comprehensive evaluation of urinary system by providing information about its morphology and function.

The present study was performed to evaluate the utility of MRU in functional assessment of urinary system in children, by comparing the measurements of differential renal function (DRF) with the results of dynamic renal scintigraphy (DRS).

As opposed to foregoing research in which renal scintigraphy was performed mainly using 99mTc-mercaptoacetyltriglycine (99mTc-MAG3), in our study DRS was performed with technetium-99 m-ethylenedicysteine (99mTc-EC). Moreover application of F -5 technique in our MRU protocol (meaning that furosemide was injected 5 min before gadolinium based contrast agent) enabled to perform examination without urinary bladder catheterization and made it less invasive.

## Materials and methods

The study was conducted in The Children’s Memorial Health Institute between 2019 and 2021. The cohort consisted of 46 patients aged 1 week to 17 years (median 7 (0.5; 13) years, 17 (37%) girls, 29 (63%) boys) who underwent dynamic renal scintigraphy due to various clinical reasons including pelvicalyceal dilatation, other CAKUT, recurrent urinary tract infections and arterial hypertension. Inclusion criteria for the MRU study included patient’s and/or legal guardian’s informed consent for the examination, estimated GFR ≥ 30 ml/min/1.73m2 (calculated by Schwartz Eq. [5]) and no contraindications for gadolinium contrast-enhanced MRI and furosemide administration. The study protocol was approved by the ethics committee of our Institute.

### Dynamic renal scintigraphy (DRS)

The DRS studies were performed using Symbia T6 and Symbia S scanners (Siemens, Erlangen, Germany). Prior to examination all patients were hydrated orally with 10 ml/kg of water. The image acquisition in posteroanterior view started simultaneously with intravenous injection of technetium-99 m-ethylenedicysteine (99mTc-EC) at a dose of 74–185 MBq depending on the body mass. Furosemide at a dose of 0.5-1 mg/kg (max. 20 mg) was administrated after 20 min of acquisition if the radionuclide continued to concentrate in the renal parenchyma. The images were analyzed by nuclear medicine specialists using dedicated software. Regions of interest were drawn manually on the heart, kidneys and perirenal background. Differential renal function was calculated using the Area Under the Curve method between 60 and 120 s after radionuclide administration. For the purpose of this study, the results of DRS were retrieved from patients’ medical history.

### Magnetic resonance urography (MRU)

All MRU examinations were performed using 1.5 Tesla Magnetom Avanto Fit scanner (Siemens, Erlangen, Germany) according to the protocol based on the guidelines developed by Khrichenko et al. (The Children’s Hospital of Philadelphia, Pennsylvania, USA) [6]. The examination lasted ca. 45–60 min. Children younger than 6 years old were examined in sedation or general anesthesia except for newborns and infants below 3 months old who were examined in natural sleep (*feed-and-sleep* technique).

In order to provide proper distension of the urinary tracts and prevent excessive concentration of gadolinium contrast agent in the urine, participants were hydrated intravenously one hour prior to examination (without sedation: 10 ml/kg, with sedation: 4 ml/kg for first 10 kg, 2 ml/kg for second 10 kg and 1 ml/kg for every kg above 20 kg) [7]. In our study children were not catheterized. Therefore to prevent bladder overfilling and premature termination of the examination, hydration was limited to 250 ml and those children who controlled their bladder were asked to urinate directly before the study. Furosemide was administered intravenously at a dose of 1 mg/kg (max. 20 mg) 5 min before contrast agent injection. Gadolinium based contrast agent was administered at a dose of 0.2 ml/kg (0.1 mmol/kg, max. 20 ml) in a slow intravenous injection with injection rates between 0.1 and 0.4 ml/s.

Functional analysis was based on pre- and post-contrast dynamic T1 sequences – VIBE (*volumetric interpolated breath-hold examination*, Siemens, Erlangen, Germany*)* obtained in the coronal plane covering the kidneys and the bladder. Pre-contrast images were used to establish initial signal intensity. Post-contrast dynamic T1 sequence consisted of 50 dynamic scans obtained with increasing intervals within 15 min.

Functional analysis of dynamic images was performed using dedicated external software (available for free at: https://www.parametricmri.com/ ) developed by The Children’s Hospital of Philadelphia (Philadelphia, Pennsylvania, USA): CHOP-fMRU – by one radiologist and pMRI – twice by one radiologist and once by second radiologist. Differential renal function was calculated basing on volume of enhancing renal parenchyma and the Rutland-Patlak method (vpDRF) without prior knowledge of DRS results. pMRI results were used to assess intra- and interobserver agreement.

### Statistical analysis

In the descriptive statistics for nominal variables the number and percentage of occurrences of a given category was reported, and for continuous variables, the normality of the distribution of the variable was first tested using the Shapiro-Wilk test, then for normally distributed variables, the arithmetic mean and standard deviation (SD) were given along with the minimum-maximum range, and for variables with a distribution deviating from the normal, the median along with 25% and 75% quantiles (Q1, Q3) and a minimum-maximum range was reported.

The Pearson correlation, intraclass correlation (ICC, a two-way model for agreement), and the Bland-Altman method were used to assess the agreement of the results of CHOP-fMRU and pMRI methods versus the renal scintigraphy, to assess the repeatability of the pMRI method, and to compare CHOP-fMRU and pMRI methods. The assessment of the agreement between methods of the measurement of differential renal function was performed for the data for the right kidney. The results were also presented graphically using scatterplots and Bland-Altman plots.

A statistical significance level was set at 0.05 in all analyzes. The analysis was performed in the R statistical package, version 3.6.3 (R Core Team (2020). R: A language and environment for statistical computing. R Foundation for Statistical Computing, Vienna, Austria. URL https://www.R-project.org/.).

## Results

The cohort of our study consisted of 46 patients aged 1 week to 17 years (median 7 (0.5; 13) years, 17 (37%) girls, 29 (63%) boys). The interval between DRS and MRU studies did not exceed 6 months, in which no urinary tract infection or surgical interventions were reported. The most common indication for examination was pelvicalyceal dilatation. All indications are shown in Table 1.

**Table 1:** Indications for functional imaging

Indication\Age	0-1y/o	1–5 y/o	6–10 y/o	11–15 y/o	16–18 y/o	Total (N = 46)
Pelvicalyceal dilatation	4	5	4	5	4	22 (47.8%)
Pelvicalyceal duplication	5				1	6 (13.0%)
Polycystic kidney disease	2		2			4 (8.7%)
Single renal cyst	1		1	1		3 (6.5%)
Horseshoe kidney		1		1		2 (4.3%)
Arterial hypertension				2		2 (4.3%)
Urinary incontinence			1	1		2 (4.3%)
Abnormal renal structure			1			1 (2.2%)
Tuberous sclerosis					1	1 (2.2%)
Urinary bladder dysfunction					1	1 (2.2%)
Policystic dysplastic kidney	1					1 (2.2%)
Bladder exstrophy	1					1 (2.2%)

The results of differential renal function (DRF) measurements based on DRS and MRU examinations are presented in Table 2.

**Table 2:** Evaluation of differential renal function in both methods - descriptive statistic

Differential renal function	*N* = 46
**DRS – right kidney [%]**	
Median [Q1, Q3]	48.5 [43.0, 56.2]
Min-Max	24, 100
**DRS – left kidney [%]**	
Median [Q1, Q3]	51.5 [43.8, 57.0]
Min-Max	0, 76
**MRU - CHOP – right kidney [%]**	
Median [Q1, Q3]	49.0 [45.0, 58.0]
Min-Max	22, 100
**MRU - CHOP – left kidney [%]**	
Median [Q1, Q3]	51.0 [42.0, 55.0]
Min-Max	0, 78
**MRU - pMRI – right kidney [%] (reader 1, reading 1)**	
Median [Q1, Q3]	49.0 [43.2, 56.5]
Min-Max	14, 100
**MRU - pMRI – left kidney [%] (reader 1, reading 1)**	
Median [Q1, Q3]	51.0 [43.5, 56.8]
Min-Max	0, 86
**MRU - pMRI – right kidney [%] (reader 1, reading 2)**	
Median [Q1, Q3]	49.5 [43.2, 56.0]
Min-Max	14, 100
**MRU - pMRI – left kidney [%] (reader 1, reading2)**	
Median [Q1, Q3]	50.5 [44.0, 56.8]
Min-Max	0, 86
**MRU - pMRI – right kidney [%] (reader 2, reading 3)**	
Median [Q1, Q3]	49.5 [40.5, 58.0]
Min-Max	9, 100
**MRU - pMRI – left kidney [%] (reader 2, reading 3)**	
Median [Q1, Q3]	50.5 [42.0, 59.5]
Min-Max	0, 91

### Concordance between DRS and MRU in DRF measurements

To assess the concordance between the two methods the results for the right kidney were analyzed (the results for the left kidney are a complement to 100%) basing on Reader 1 measurements.

Differences in DRF calculated by CHOP-fMRU and pMRI in regard to DRS are shown in Table 3.

**Table 3:** Differences between DRF results based on MRU vs. DRS

Parameter	*N* = 46
**Difference between DRF results measured in CHOP-fMRU vs. DRS [p.p.]**	
Mean (SD)	0.4 (3.0)
Median [Q1, Q3]	0.0 [-1.0, 2.0]
Min-Max	-10, 6
**Difference between DRF results measured in pMRI (reading 1) vs. DRS [p.p.]**	
Mean (SD)	-0.2 (4.8)
Median [Q1, Q3]	0.0 [-1.0, 2.8]
Min-Max	-20, 7

**Fig. 1:**
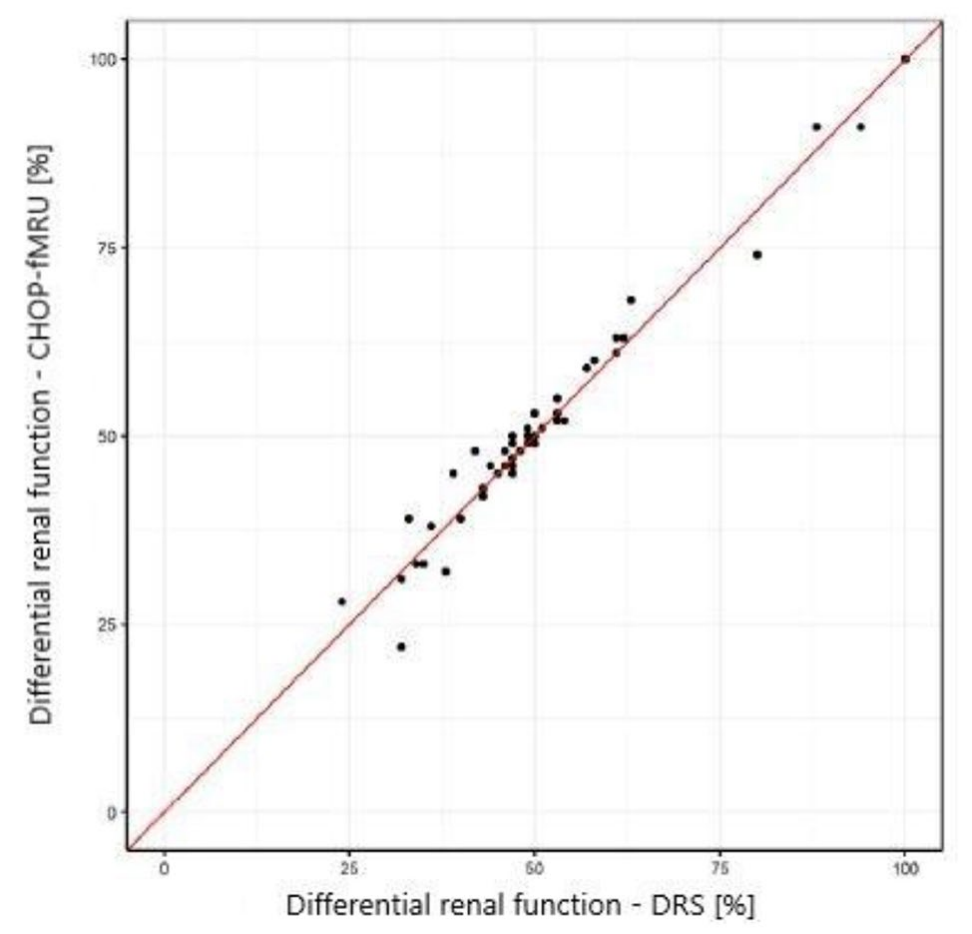
Scatterplot comparing DRF measurement in CHOP-fMRU and DRS

**Fig. 2:**
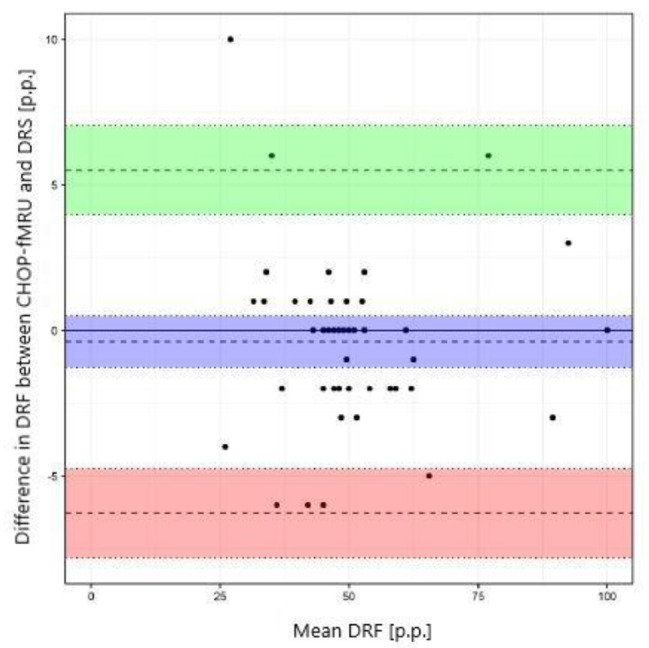
Bland–Altman plot comparing DRF measurement in CHOP-fMRU and DRS

**Fig. 3:**
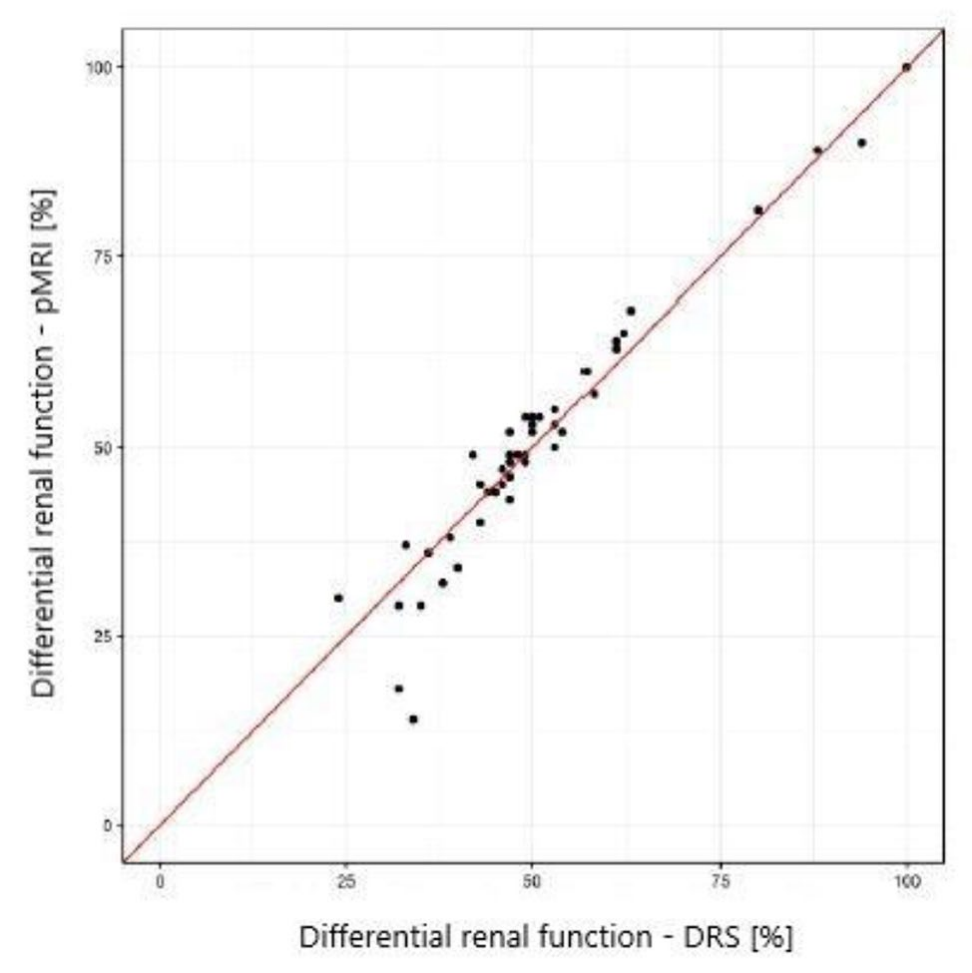
Scatterplot comparing DRF measurement in pMRI and DRS

**Fig. 4:**
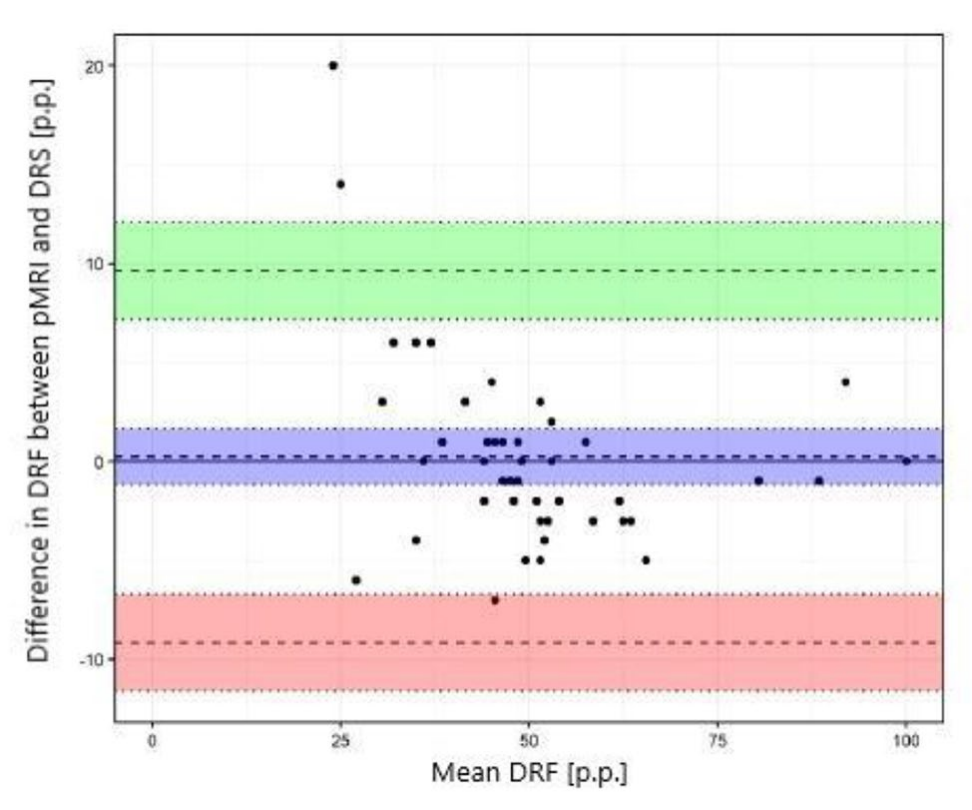
Bland–Altman plot comparing DRF measurement in pMRI and DRS

The study showed excellent concordance between the measurements of differential renal function in DRS and both MRU programs with Pearson correlation coefficient 0.987 (*p*-value < 0.001) for CHOP-fMRU and 0.971 (*p*-value < 0.001) for pMRI. Intraclass correlation coefficient (ICC, two-way model for agreement) between the two methods was 0.987 (95% CI 0.976–0.993, *p*-value: <0.001) and 0.969 (95% CI 0.945–0.983, *p*-value < 0.001) respectively. The good concordance between DRS and MRU was also depicted in the Bland-Altman plots with 95% limits of agreement − 6.29–5.50 p.p. for CHOP-fMRU and − 9.15–9.63 p.p. for pMRI. Visual analysis of the Bland-Altman plots shows higher differences of the results in patients with significant relative impairment of one kidney’s function. The scatterplots and Bland-Altman plots comparing DRF measurements in MRU and DRS are depicted on Figs. 1, 2, 3 and 4.

Assuming that normal differential renal function ranges between 45% and 55%, the qualitative results of MRU obtained using CHOP-fMRU were discordant with DRS in three patients. In these cases DRS revealed mild relative impairment of one kidney’s function while CHOP-fMRU results were normal. Regarding pMRI results the discrepancies concerned two patients with mild relative impairment of one kidney’s function in DRS and normal pMRI results and one patient with normal DRS results and mild relative impairment of one kidney’s function in pMRI.

### Intraobserver agreement

**Fig. 5:**
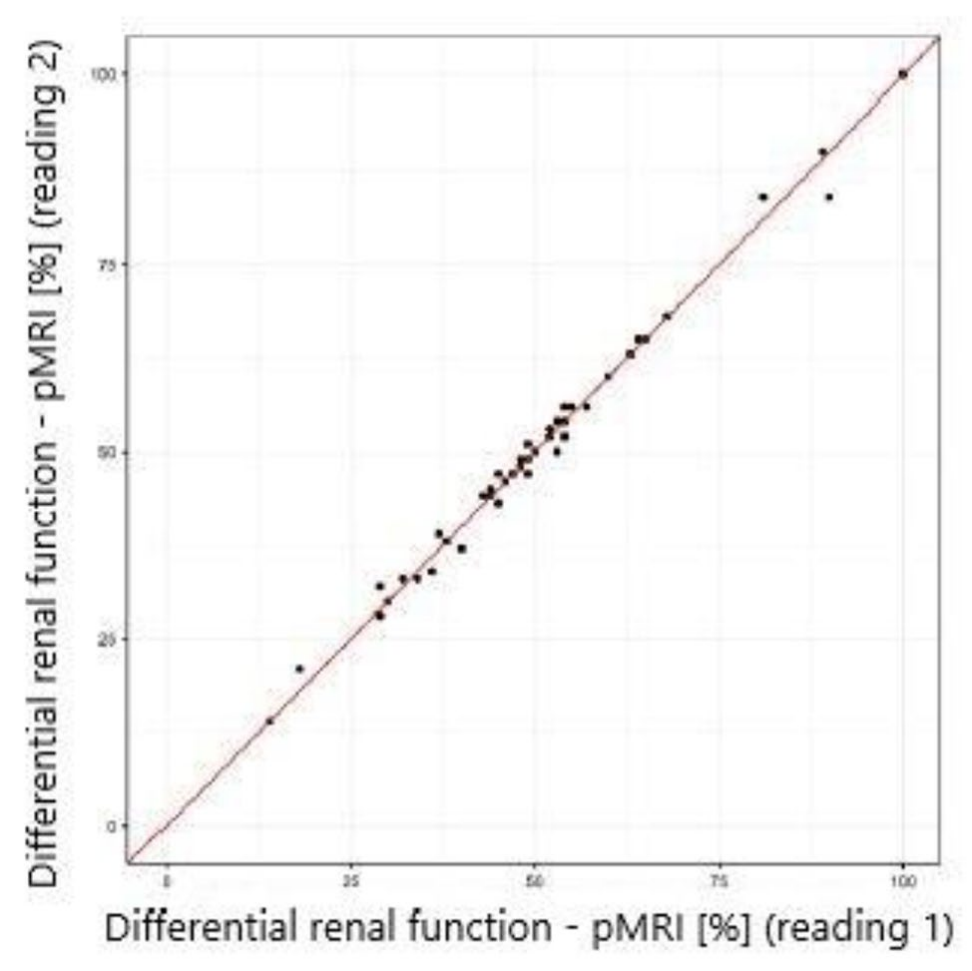
Scatterplot comparing DRF measurement in pMRI – reading 1 and 2 (intraobserver agreement)

**Fig. 6:**
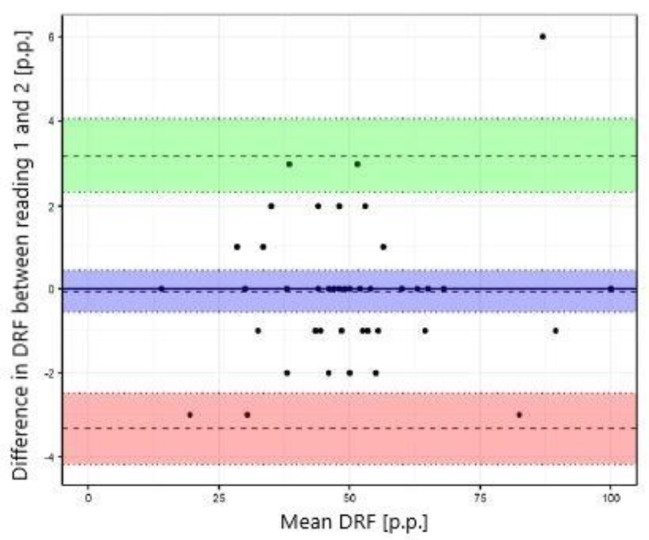
Bland–Altman plot comparing DRF measurement in pMRI – reading 1 and 2 (intraobserver agreement)

Intraobserver agreement was assessed for DRF measurements calculated using pMRI software. Statistical analysis showed excellent intraobserver agreement with mean difference 0.1 ± 1.7 p.p. and ICC 0.996 (95% CI 0.994–0.998), *p*-value < 0.001. High repeatability was depicted by the scatterplot (Fig. 5) and the Bland-Altman plot with 95% limits of agreement − 3.33–3.20 p.p. (Fig. 6).

### Interobserver agreement

**Fig. 7:**
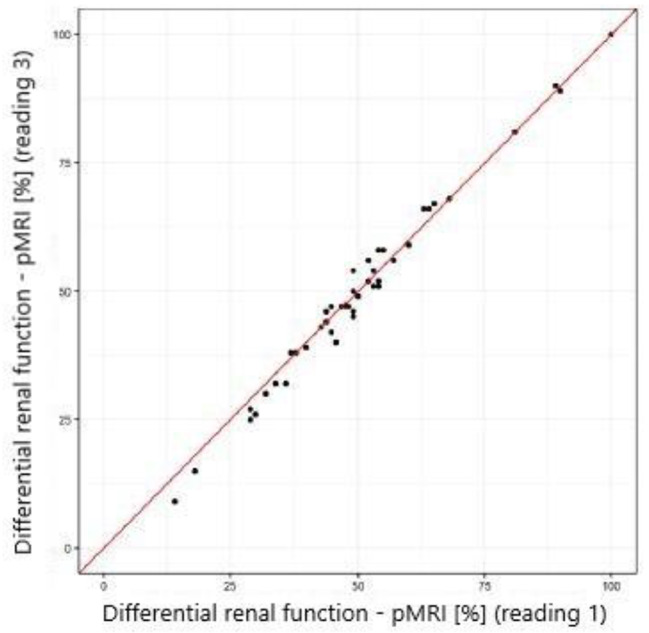
Scatterplot comparing DRF measurement in pMRI – reading 1 and 3 (interobserver agreement)

**Fig. 8:**
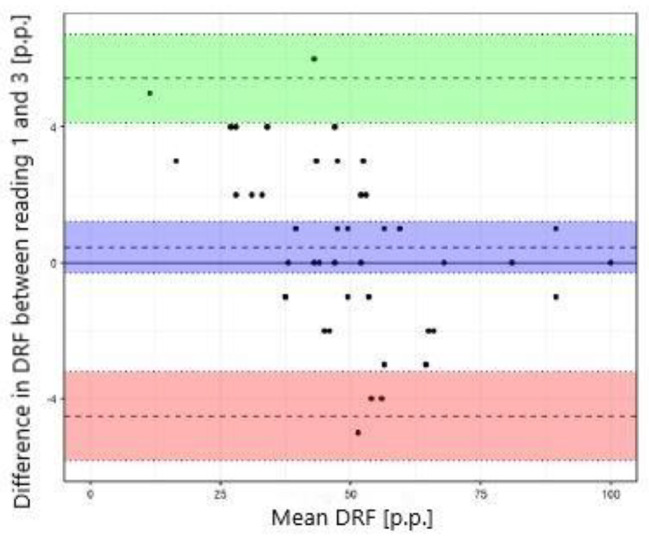
Bland–Altman plot comparing DRF measurement in pMRI – reading 1 and 3 (interobserver agreement)

Interobserver agreement between two radiologists was assessed for DRF measurements calculated using pMRI software. Statistical analysis showed excellent interobserver agreement with mean difference − 0.5 ± 2.5 p.p. and ICC 0.992 (95% CI 0.986–0.996), *p*-value < 0.001. High repeatability was depicted by the scatterplot (Fig. 7) and the Bland-Altman plot with 95% limits of agreement − 4.51–5.43 p.p. (Fig. 8).

## Discussion

The functional assessment of urinary system including evaluation of differential renal function constitutes an important part of diagnostic process in pediatric nephrology and urology. Currently, dynamic renal scintigraphy is considered a gold standard for renal function evaluation in children. Our study conducted with participation of 46 pediatric patients, who underwent both DRS and MRU examinations, shows that DRF can be reliably assessed using MRU with very good concordance with DRS and excellent intra- and interobserver agreement.

These results are consistent with foregoing research comparing these two methods in both pediatric and adults patients [8–17].

The most extensive multicentric study, in which MRU and renal scintigraphy (RS) were compared, was performed by Claudon et al. [10] and involved 295 patients in 14 university hospitals. In this research both adults and children were included, however vast majority of participants (*n* = 220) was below 2 years old. This study concerned only the patients suspected of urinary obstruction and proved the equivalence of MRU and RS for measurement of DRF in patients with moderately dilated pelvicalyceal system. In patients with severely dilated pelvicalyceal system the mean DRF measurement calculated using MRU was underestimated by 4% in regard to RS results.

Teh et al. [17] conducted a comparative study among patients with various disorders of urinary system observing good correlation between MRU and RS results with Pearson’s correlation coefficient 0.97 (*p* < 0.001) however the analysis included only 19 adult patients (aged 16–83 years old).

The study conducted by Damasio et al. [9] involved 52 pediatric patients (aged 0 to 19 years old, including 17 infants) with dilated pelvicalyceal system. Although it also showed good correlation between the two methods, obtained correlation coefficient was lower (Spearman’s correlation coefficient 0.56, *p* < 0.005).

Grattan-Smith et al. [12] compared DRF measured using MRU and RS in 39 children aged 1 month to 14 years old (mean age 1.4 years old) with unilateral pelvicalyceal dilatation and obtained correlation coefficient 0.98. Similar results with correlation coefficient 0.93 were obtained by Perez-Brayfield et al. [13] who compared DRF measurements in 71 patients aged 1 month – 17 years old. Genseke et al. [14] analyzed the DRF results of 30 pediatric patients suspected of urinary obstruction, dividing them into two age groups: A – below 2 years old and B – aged 2–17 years old. The correlation coefficient was 0.94 and 0.97 respectively.

Monocentric studies with participation of children (1 month – 17 years old) comparing DRF measurements using MRU and RS were also conducted by Dzananovic et al. [8], Rohrschneider et al. [11], Boss et al. [15] and Reither et al. [16] proving the high concordance of the results.

As opposed to the previously conducted studies, we compared MRU results with renal scintigraphy performed using 99mTc-ethylenedicysteine (99mTc-EC). In all adduced studies renal scintigraphy was performed using 99mTc-mercaptoacetyltriglycine (99mTc-MAG3). Furthermore, in some patients Claudon et al. [10] used 99mTc-diethylenetriaminepentaacetic acid (99mTc-DTPA) and Grattan-Smith et al. [12] and Perez-Brayfield et al. [13] − 99mTc-DTPA and 99mTc-dimercaptosuccinic acid (99mTc-DMSA). 99mTc-MAG3 is the radiotracer most commonly used for dynamic renal scintigraphy [18]. In comparison to 99mTc-MAG3, the renal clearance of 99mTc-EC is higher while the plasma protein biding is significantly lower, which results in a larger volume of distribution [19–21]. The advantages of 99mTc-EC over 99mTc-MAG3 include also lower hepatobiliary uptake and easy labeling of tracer at room temperature [20–22]. Our study proved that concordance between MRU and renal scintigraphy is equally good regardless of used radionuclide including 99mTc-EC.

The other difference in our protocol regards the time of furosemide administration during MRU. While most researchers use F-15 (administration of furosemide 15 min prior to contrast agent injection) [12, 17] or F0 (administration of furosemide right before contrast agent injection) [9] technique, we adopted F-5 method with furosemide administration 5 min before contrast agent injection. This approach provides enough time for furosemide to develop its diuretic effect before dynamic MR sequence acquisition and additionally enables to conduct the rest of the examination without bladder catheterization.

Some authors advise urinary bladder catheterization to minimize the effect of filled bladder on contrast excretion into urinary tract, prevent vesicoureteral reflux and reduce the risk of disruption the examination due to patient’s need to void [6, 7, 23]. Similarly to Vivier et al. [24], Teh et al. [17] and Rohrschneider et al. [11] we decided to abandon this procedure to make the examination less invasive. In order to minimize bladder overdistension and patients’ discomfort during the examination, we asked patients to void directly before MRU and introduced F -5 technique. This procedure did not have a negative effect on the results of the study which remained similar to the research performed with bladder catheterization [12, 13]. 

The results of our study confirm a strong concordance between DRS and MRU in measuring differential renal function. For 85% of the results obtained in both MRU software programs (CHOP-fMRU and pMRI) the difference in regard of DRS measurements did not exceed 5 p.p. The most significant differences (exceeding 5 p.p.) occurred in patients with massive pelvicalyceal dilatation and significant parenchymal loss. Similar observations were made by other authors [10, 12–14, 17]. Claudon et al. [10], who conducted the most extensive comparative study, proved the equivalence of MRU and RS for measurement of DRF in patients with moderately dilated pelvicalyceal system, while in patients with severely dilated pelvicalyceal system the mean DRF calculated using MRU was underestimated by 4% in comparison to RS. The authors suggested several reasons of these discrepancies: poorer reproducibility of RS results in patients below 6 months old or with low glomerular filtration rate, difficulty with drawing ROI in the presence of severely dilated pelvicalyceal system and a partial volume effect in MRU due to sampling in a very thin parenchymal ROI. The studies by Grattan-Smith et al. [12] and Perez-Brayfield et al. [13] showed similar discrepancies between the methods in patients with massive dilation of collecting systems and significant parenchymal loss. They considered MRU results to be more accurate than DRS due to better contrast and spatial resolution allowing more reliable separation of renal parenchyma from the background and dilated pelvicalyceal system. Teh et al. [17] obtained the difference in DRF measurements up to 10 p.p. in cases of hydronephrosis with thinned cortex. The authors attributed it to capacious renal pelvis which may attenuate radionuclide intensity in DRS. Moreover, they indicated that in a small kidney even minor inaccuracy in the ROI area may result in a significant percentage of difference in volume measurement and therefore affecting overall DRF. We share the opinion that MRU results are more accurate in those patients and correspond better to clinical features.

Our study also shows excellent intra- and interobserver agreement in DRF calculations based on MRU (using pMRI software). Our results in this regard are concordant with other research [9–11]. Rohrschneider et al. [11] assessed interobserver reliability for DRF measurements with the intraclass correlation coefficient (ICC) 0.932. Damasio et al. [9] obtained high correlation coefficient for intraobserver repeatability in both DRS and MRU methods and interobserver repeatability in MRU. In the study by Claudon et al. [10] the assessment of reproducibility for intra- and interobserver agreement was conducted for a random sample of examinations and was substantial for both techniques. The ICC for intraobserver agreement in DRS was 0.9 and MRU 0.81, while for interobserver agreement 0.75 and 0.76 respectively.

In our analysis the agreement between DRS and MRU results is comparable for both programs: CHOP-fMRU and pMRI. However, shorter and more user-friendly post-processing advocate the use of pMRI.

A major disadvantage of MRU is its long duration (approximately 45–60 min), which causes the necessity for sedation in younger children (usually below 6 years old) [23, 25]. Furthermore, the relatively high cost and low availability of MR may limit more common application of this imaging technique [26]. 

### Strengths and limitations

Strengths of our study include a wide range of pediatric population (1 week to 17 years old) and variety of clinical indications to functional evaluation of the urinary system. Moreover, our research differs from foregoing studies in radionuclide used to perform renal scintigraphy. We also modified MRU protocol in regard of furosemide administration to avoid bladder catheterization. In addition our study include the assessment of intra- and interobserver agreement.

The major limitation of our study is relatively small group of patients as it involves single center experience only. Renal scintigraphy examinations were assessed by different nuclear medicine specialists and the results were retrieved from patients’ medical history. Therefore the intra- and interobserver agreement was not assessed for this technique. Furthermore, there is no objective noninvasive method that could settle which DRF measurements are more accurate in cases of higher discrepancies.

## Conclusions

According to our study, there are no significant differences between magnetic resonance urography and dynamic renal scintigraphy performed with 99mTc-EC in calculating differential renal function. MRU should become a significant part of diagnostic imaging in children as it provides excellent morphological and simultaneously functional evaluation of urinary system without exposure to ionizing radiation. Therefore, MRU contributes to earlier diagnosis, facilitates clinical decisions, supports planning surgical intervention and enables the treatment effectiveness assessment. In conclusion, MRU is a safe imaging technique that could take an important place in diagnostic algorithm in pediatric urology.

## Data Availability

The datasets generated during the current study are available from the corresponding author on request.
